# Use of public water supply fluoride concentration as an indicator of population exposure to fluoride in England 1995–2015

**DOI:** 10.1007/s10661-020-08304-3

**Published:** 2020-07-14

**Authors:** David J. Roberts, J. Morris, A. Wood, N. Q. Verlander, G. S. Leonardi, T. Fletcher

**Affiliations:** 1 Field Epidemiology Training, Public Health England, Colindale, London, UK; 2 European Programme for Interventional Epidemiology Training (EPIET), European Centre for Disease Prevention and Control, Stockholm, Sweden; 3 Environmental Epidemiology, Centre for Radiation Chemical and Environmental Hazards, Public Health England, Chilton, Oxfordshire, OX11 0RQ UK; 4 University of Birmingham School of Dentistry, Edgbaston, Birmingham, UK; 5 Health Intelligence, Public Health England, Birmingham, UK; 6 Statistics, Modelling and Economics Department, Public Health England, Colindale, London, UK; 7 Department of Social and Environmental Health Research, London School of Hygiene and Tropical Medicine, London, UK

**Keywords:** Exposure assessment, Fluoride, Fluoridation, Surveillance, Monitoring

## Abstract

Public health monitoring of Community Water Fluoridation (CWF) schemes requires estimates of exposure to fluoride in public water supplies (PWS). We aimed to use routine data to estimate population exposure to PWS-fluoride in England and to determine whether PWS-fluoride exposure from 2005 to 2015 could be used as a proxy for exposure for 1995–2004, when fluoride concentration data that could be linked to population health data were unavailable. We calculated annual mean water supply zone PWS-fluoride concentrations from monitoring data for 1995–2015, stratified by fluoridation scheme-flagging. We allocated annual 2005–2015 mean PWS-fluoride concentrations to small area boundaries to describe population exposure within five concentration categories (< 0.1 to ≥ 0.7 mg/L). We compared zone-level 1995–2004 and 2005–2015 mean PWS-fluoride concentrations using Spearman correlation. Most (72%) of the population received PWS with < 0.2 mg/L fluoride and 10% with ≥ 0.7 mg/L. Fluoride concentrations in 1995–2004 and 2005–2015 were similar (median 0.11 mg/L (lower quartile–upper quartile (LQ–UQ) 0.06–0.17) and 0.11 mg/L (LQ–UQ 0.07–0.17), respectively) and highly correlated (coefficient 0.93) if un-fluoridated but differed (1995–2004 median 0.78 mg/L (LQ–UQ 0.59–0.92); 2005–2015 0.84 mg/L (LQ–UQ 0.72–0.95)) and correlated weakly (coefficient 0.31) if fluoridated. Fluoride concentrations in 2005–2015 approximate those in 1995–2004 but with a greater risk of misclassification in fluoridation schemes.

**Electronic supplementary material:**

The online version of this article (10.1007/s10661-020-08304-3) contains supplementary material, which is available to authorized users.

## Introduction

The fluorine element and fluoride compounds (henceforth referred to simply as ‘fluoride’) are naturally occurring and likely to be found in sources of drinking water in varying amounts and are also present in some foods and drinks. Exposure to fluoride can reduce the risk of dental caries (tooth decay) (Selwitz et al. 2007), and Community Water Fluoridation (CWF) schemes that adjust fluoride concentrations in water supplies to target concentrations typically in the range of 0.7–1 mg/L have been shown to effectively reduce caries prevalence and severity in children (Iheozor-Ejiofor et al. 2015). In some parts of England, as a result of the geology of the area, fluoride concentrations in public water supplies (PWS) already reach the UK target concentration for CWF schemes (1 mg/L). In other areas that are part of fluoridation schemes, the fluoride concentration has been adjusted to reach this concentration. Currently, around 10% of the England population (six million people) live in areas with fluoridation schemes where the concentration has been adjusted.

In addition to the known benefits, harmful health effects have been attributed to fluorides; convincing evidence of a causal association with these at the levels permitted by water quality legislation is lacking, though an increase in dental fluorosis has been identified (Iheozor-Ejiofor et al. 2015). Current legislation in England (Statutory Instrument 2018 no. 647 2018) allows for up to 1.5 mg/L of fluoride to be present in PWS which mirrors EU legislation and is intended to be protective against any harmful effects from chronic exposure, including dental fluorosis which might be unsightly. In England, Public Health England (PHE) monitors the health effects of the adjustment of PWS fluoride concentrations for fluoridation schemes on behalf of the Secretary of State for Health and Social Care and in line with legislation (Water Industry Act 1991 c.56 1991). Previous monitoring (Public Health England 2014) and other epidemiological studies (McLaren and Emery 2012; Skinner 2012) have used data from routine PWS monitoring to estimate population exposure to fluoridation. However, these population exposure models were limited to simple binary exposures (i.e. fluoridated or not) rather than the PWS fluoride concentration, risking exposure misclassification and preventing dose–response analysis. The latter may be important when determining the optimal fluoride concentration to maximise caries prevention benefit and minimise dental fluorosis and also to consider evidence for causal associations with health effects for which evidence of an association is less established. Linkage of fluoride PWS concentration data with health data to assign exposure typically requires geo-referencing of PWS monitoring data onto administrative boundaries. Therefore, exposure models may also be limited by constraints in availability of geo-referenced routine monitoring data for certain time periods, meaning assumptions may have to be made about exposure in these periods. Quantification of past population exposures may be important when investigating potential associations between fluoride exposure and caries development in older children (as incorporation of fluoride into developing tooth tissue and after tooth eruption are both likely to play a role in modifying caries risk) (Singh and Spencer 2004). Additionally, quantifying prior exposure may also be useful for investigating more recently occurring health outcomes with longer induction periods, such as some cancers (Checkoway et al. 1990).

We aimed to estimate population exposure to increasing categories of fluoride concentration in PWS in England to use as an exposure indicator in public health monitoring of water fluoridation schemes for the 2018 PHE fluoridation health monitoring report (Public Health England 2018). We further aimed to determine whether contemporary (2005–2015) routine fluoride concentration monitoring data could be used as a proxy indicator of population exposure for prior years when geo-referenceable data was not easily available.

## Methods

### Water fluoridation schemes in England

PWS are delivered through distinct water supply zones (WSZs). Each WSZ is defined by either a single point of water supply or, if there are multiple water supply sources of a similar nature and treatment, WSZs are ascribed to permanent resident populations of 100,000 or fewer (Drinking Water Inspectorate 2016b). The number and size of WSZs are reviewed annually by water undertakers and the Drinking Water Inspectorate (DWI, the water quality regulator for England and Wales) and, from 2004, the permitted number of residents per WSZ was increased which contributed to a subsequent reduction in the number of WSZs (forming larger zones with greater populations).

Water companies have a duty to monitor the fluoride concentration of PWS in all of the WSZs they supply and provide these monitoring data to the DWI. In addition to the upper limit, prescribed concentration value (PCV) of 1.5 mg/L fluoride concentrations in fluoridated areas should reach the 1.0 mg/L target (Drinking Water Inspectorate 2016a). Sampling points to monitor fluoride concentrations within the WSZs are randomly chosen (typically consumers’ taps) and must be representative of the WSZ as a whole (Statutory Instrument 2018 no. 647 2018). Samples may also be taken from ‘water supply points’, such as service reservoir outlets, treatment works outlets or blending points. These supply points may supply more than one zone, provided there is no substantial difference in the concentration between the supply point and consumers’ taps in the zone (Drinking Water Inspectorate 2016a). Sampling frequency is determined by WSZ factors such as population size and daily volume of water supplied (for supply point samples) (Statutory Instrument 2018 no. 647 2018). Regulations establish mandatory minimum standards for accuracy and precision of fluoride concentration testing (Statutory Instrument 2018 no. 647 2018). The limit of detection must be within 10% of the prescribed concentration (1.5 mg/L), which would therefore be 0.15 mg/L. However, this is only a minimum standard which we hypothesised real test performance would outperform, because a prior description of water fluoride concentrations in England and Scotland revealed 48% of areas had fluoride concentrations < 0.1 mg/L (with a minimum value of 0.04 mg/L) (Blakey et al. 2014). Though the DWI has maintained routine fluoride PWS monitoring data since 1995, digitised geographic WSZ-boundary data (shapefiles) have only been collated since 2004.

### Rationale for using PWS fluoride concentration as a proxy for total fluoride intake in population exposure models

Fluoride is readily and predictably absorbed into the body via the gastrointestinal tract, and this is the main mode of absorption (International Programme on Chemical Safety (Environmental Health Criteria 227) 2002). Thus, fluoride in drinking water is generally bioavailable. This is unlikely to be affected by water hardness at concentrations of around 1 mg/L (Maguire et al. 2005). Available evidence, though limited in extent, strongly suggests, in terms of chemistry and bioavailability, there is no important difference between added and ‘natural’ fluoride occurring from geological sources (Maguire et al. 2005; Jackson et al. 2002). Drinking water with more than 0.3 mg/L of fluoride is amongst the main sources in human total fluoride intakes, particularly at higher fluoride concentrations (e.g. > 0.7 mg/L) typically seen in fluoridated supplies (‘Opinion on critical review of any new evidence on the hazard profile, health effects, and human exposure to fluoride and the fluoridating agents of drinking water’ 2011; Zohouri et al. 2006a). Fluoride water concentrations therefore correlate with human biomarkers of exposure such as urine (Till et al. 2018; Zipkin et al. 1956) and blood plasma or serum (Rafique et al. 2012). For example, maternal urinary fluoride (MUF) concentrations, used as a biomarker of fluoride intake, were almost twice as high in pregnant women living in fluoridated communities than those in non-fluoridated communities in Canada (Till et al. 2018). Log MUF concentrations increased linearly with increasing water fluoride concentration, which was the strongest predictor of urinary fluoride concentration (Till et al. 2018). This study relied upon spot urinary fluoride measurements; these have been validated against 24 h urinary fluoride measurement (recommended for definitive estimation) in children (Zohouri et al. 2006b), but not extensively used in adults. However, there was consistency between repeated spot samples (Till et al. 2018). Living in communities with CWF has also been demonstrated to be a strong determinant of spot urinary fluoride concentrations in the 3- to 79-year-old population in Canada on a population survey, with age having little effect, supporting the validity of spot urinary assessment across age groups and the use of CWF as a proxy for total fluoride intake (McLaren 2016). Finally, the prevalence and severity of dental fluorosis (a relatively specific indicator of increased fluoride consumption) are higher amongst children and young people in fluoridated regions (Pretty et al. 2016; Beltran-Aguilar et al. 2010; Iheozor-Ejiofor et al. 2015), and dental caries prevalence and severity lower (Public Health England 2018; Iheozor-Ejiofor et al. 2015), meaning there is some face validity to using PWS fluoride concentrations as a proxy for total intake.

Some potential limitations to our rationale should also be considered. Alternative fluoride sources, for example from tea or fluoridated dentifrices, may also be important determinants of total fluoride at high intakes (particularly dentifrices in children (Zohouri et al. 2013)), but their relative contribution to total fluoride intake is generally lower when water fluoride concentrations reach levels typically targeted by fluoridation schemes (‘Opinion on critical review of any new evidence on the hazard profile, health effects, and human exposure to fluoride and the fluoridating agents of drinking water’ 2011; Zohouri et al. 2006a). Additionally, globalised production and importation of foodstuffs may modify fluoride intake independent of local water fluoride concentration (a phenomenon referred to as the ‘halo effect’) (Griffin et al. 2001). This would presumably be of most relevance to population groups who consume more manufacturer-added than consumer-added water such as from soft drinks in children. For example, estimates of water intake in 11–12 years olds in North East England estimated carbonated drinks provided 17% of water intake (Zohouri et al. 2004). Even so, consumer-added water still accounted for a much higher percentage of total water intake (35%) than manufacturer-added water (24%), meaning water fluoridation is still highly likely to be a major determinant of total fluoride intake (and is supported by recent biomarker studies in children and adults referenced above). However, we recognise that the validity of PWS fluoride concentration as a proxy for total intake may vary by differing dietary habits and age groups.

### Data sources and management

Data cleaning, management and analysis were executed in Microsoft Excel 2010 (Microsoft, USA) and STATA version 14 (StataCorp, USA). Geo-referencing was performed using ArcGIS ArcMap version 10.2 (Esri, USA). Exposure indicators were estimated by combining fluoride concentration obtained from routine fluoride monitoring data from 1995 to 2015, provided by the DWI and population data obtained from the Census and related mid-year estimates computed by the Office for National Statistics (ONS). The DWI supplied copies of water company WSZ boundary files in digital format for 2004–2015, of which we were able to prepare 2005–2015 for analyses. We used Geographic Information Systems (GIS) point-in-polygon (PIP) methods to assign statistical areas to WSZ boundaries using the population weighted centroid (Chapter 7—Using GIS for Environmental Exposure Assessment: Experiences from the Small Area Health Statistics Unit 2004). The smallest geographical unit of analysis was the 2011 Lower layer Super Output Area (LSOA); analyses at larger geographical areas were performed by using LSOA level fluoride, health and population data as ‘building blocks’, aggregated to form their larger ‘parent’ 2011 Middle layer Super Output Area (MSOA) and 2011 Lower Tier Local Authority (LTLA) areas (with which their borders match). See Supplementary Table 1 for population and count information on these geographic units. The population-weighted centroid of each LSOA (‘point’), which assigns a single geographic point to each LSOA based on the largest aggregation of its population, was overlaid onto WSZs (‘polygons’), thereby allocating a LSOA (and their populations) to a WSZ. As WSZs may be aggregated or dis-aggregated to form new WSZs, the number and geographic boundaries of WSZs are not fixed over time, though this occurs at (at least) annual intervals. WSZs will retain a unique identifier (consisting of the concatenated water company name and water company WSZ reference) whilst under the ownership of a single company, which can be used to track monitoring data over the time period of the WSZ’s ownership by that particular water company. To overcome the issue of WSZs changing shape and size over time, PIP analysis was repeated for each year of available (mapped) WSZ data (2005–2015). The linked LSOA-WSZ pairs were then linked with the DWI fluoride concentration and fluoridation scheme flagging dataset, using concatenated site reference and water-company coding by year to identify common WSZ years. Arithmetic mean period fluoride concentrations for the exposure period of interest were then aggregated from LSOA to higher geographic levels, weighted by the exposed population. We deliberately selected the arithmetic mean rather than other measures of central tendency less effected by extreme values as this would most accurately reflect population exposure in the whole period of interest, which would include ‘outlier’ periods.

Area-level fluoride concentration in water supply, regardless of source, was then categorised into the following: 0.0–< 0.1 mg/L, 0.1–< 0.2 mg/L, 0.2–< 0.4 mg/L, 0.4–< 0.7 mg/L and ≥ 0.7 mg/L. Such an approach was chosen to examine possible biologic gradient in the form of presence of a dose–response, while not assuming it would follow a specific model form (Rothman et al. 2008). Rather, we intended to break the range of the study exposure into categories that could be used to look for trends in the category-specific coefficients or relative risks, while avoiding a mechanical algorithm such as the percentile method, which could lack power to detect exposure effects stronger at extreme ends of the exposure scale (Greenland 1995). These categories were selected because, from international evidence, the association between fluoride concentration and decreasing caries prevalence was thought to increase linearly with increasing fluoride concentration, with reductions in dental caries prevalence tailing off above 0.7 mg/L (U.S. Department of Health Human Services Federal Panel on Community Water Fluoridation 2015). Further categorising the fluoride concentration of the population receiving water supplies with fluoride < 0.7 mg/L would give the ability to detect a dose–response, plateau, and threshold effect at higher fluoride levels. Given the relatively low water fluoride concentrations in England, the population receiving supplies at concentrations > 0.4 mg/L was thought unlikely to be large enough for division into more than two further categories and still allow meaningful examination of associations with less common health outcomes.

Missing data, outliers and unexpected values (e.g. high fluoride concentrations in zones not flagged as fluoridated, and vice versa) were investigated. Since 2006, the DWI has retained annual records that identify, via a flag, those WSZs that have fluoridation schemes. As no new CWF schemes have been initiated since 1995, flagged WSZs were considered to have been fluoridated from at least 1995 continuously to 2015, and we checked whether all zones ever fluoridated had consistent CWF flagging. Where this was not the case, we asked the DWI to check their supporting databases as to whether the zone was truly fluoridated or not and whether inconsistent flagging may have been the result of disruption in operation of fluoride plant. If there was known disruption of fluoridation, then the zone was re-flagged as being not fluoridated for the years during which fluoridation was disrupted.

### Exposure indicator descriptive analysis

The number of water supply zones, number of samples and average number of years of monitoring data per WSZ were described from 1995 to 2015 stratified by time period (1995–2004 or 2005–2015), reflecting the variation in availability of WSZ mapping data and approach to fluoridation and its monitoring over different time periods. We then described the annual mean fluoride concentrations in each zone for these two time periods, stratified by presence of a fluoridation scheme, using histograms and box-plots. Stability of fluoride concentrations within unique zones was further described by creating scatter plots and calculating Spearman rank coefficients (stratified by presence or absence of a fluoridation scheme) for the WSZ-level period mean fluoride concentrations from 1995 to 2004 compared to 2005–2015. Spearman rank coefficients were calculated due to the skewed distribution of the data in fluoridated areas. Fluoridation schemes known to have significant disruption to operation were excluded from the creation of scatter plots and correlation analysis. MSOA-level public water supply grand mean (of the annual means) fluoride concentrations for 2005–2015 and location of fluoridation schemes were then described spatially by mapping the 2005–2015 grand mean fluoride concentrations and the distribution of fluoridation schemes onto 2011 MSOA boundaries. In order to map grand mean fluoride concentrations onto MSOA boundaries, we first calculated the grand mean fluoride concentrations for each LSOA and then aggregated to MSOA level by weighting the means of each constituent LSOA by its 2005–2015 population, using ONS mid-year population estimates. We then tabulated the MSOA-level count of population supplied (taking the period average) for categorised levels of fluoride concentration in milligrams per litre.

## Results

**Fig. 1:**
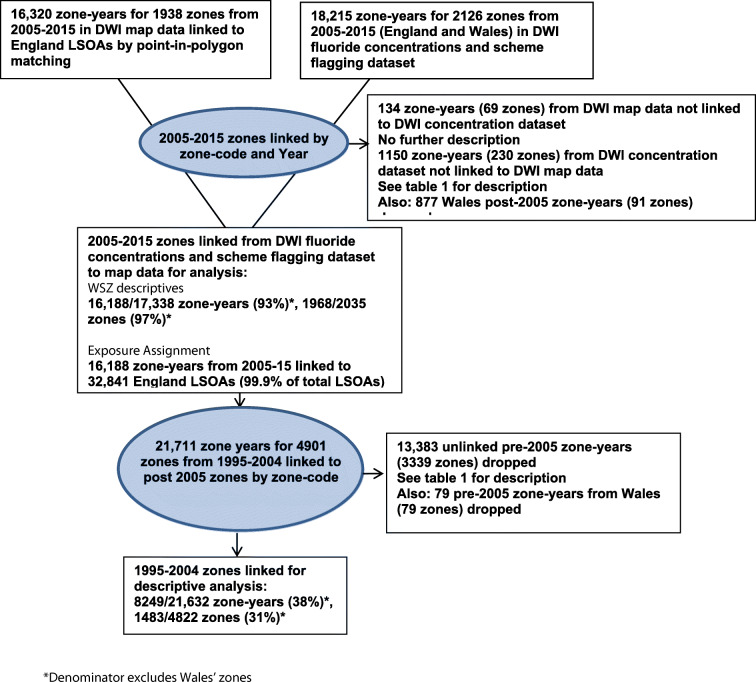
Summary of how water supply zones were selected for analysis England 1995–2015

Figure 1 summarises how zones were selected for analysis. A total of 134 zone years from 69 water supply zones were linked to LSOA geography on PIP linkage but then could not be linked to zones in the main DWI fluoride concentration dataset.^1^ No further characteristic information was available for these zones.

A further 1150 zone years from 230 zones over 2005–2015 were in the DWI fluoride concentration dataset but not linked to zone codes in the WSZ boundary files for PIP linkage. The characteristics of these zones are shown in Table 1 below.

**Table 1:** Characteristics of unlinked zone years from DWI dataset for 2005–2015 (*n* = 1150 zone years for 230 zones) and 1995–2004 (*n* = 13,383 zone years for 3339 zones)

Time period	Fluoridation scheme (%)	Median fluoride (mg/L) (LQ–UQ)	Median fluoride (mg/L) in scheme (LQ–UQ)	Median annual samples (LQ–UQ)	Median years of monitoring data per zone (LQ–UQ)
95-04	NA^a^	0.10 (0.08–0.20)	NA^a^	1 (1–1)	8 (4–9)
05-15	64 (5.6)^b^	0.10 (0.05–0.23)	0.92 (0.72–0.97)	4 (2–4)	10 (7–11)

*LQ* lower quartile, *UQ* upper quartile

^a^Not possible to assign a fluoridation scheme status

^b^A total of 1138/1150 zone years assigned a fluoridation status

The characteristics of zone years/zones that were linked between the two datasets for the time period are summarised in Table 2.

**Table 2:** Characteristics of linked zone years from DWI dataset for 1995–2004 (*n* = 8249 zone years for 1483 zones) and 2005–2015 (*n* = 16,188 zone years for 1884 zones)

Time period	Fluoridation scheme (%)	Median fluoride (mg/L) (LQ–UQ)	Median fluoride (mg/L) in scheme (LQ–UQ)	Median number of annual samples per zone (LQ–UQ)	Median years of monitoring data per zone (LQ–UQ)
95-04	491 (6.3)^a^	0.12 (0.06–0.19)	0.78 (0.57–0.90)	1 (1–6)	10 (9–10)
05-15	1566 (9.7)^b^	0.12 (0.07–0.21)	0.84 (0.66–0.94)	8 (8–9)	11 (10–11)

*LQ* lower quartile, *UQ* upper quartile

^a^A total of 7791/8249 zone years assigned a fluoridation status

^b^A total of 16,135/16,188 zone years assigned a fluoridation status

Median fluoride was slightly lower in the un-linked 2005–2015 zones, but higher if in the 5.6% in a fluoridation scheme. Fewer annual samples were taken in these un-linked zones; however, the 10-year typical duration of a zone is similar to the linked zones. Thirty-three out of 192 zones flagged as ever fluoridated from 2006 to 2015 in the DWI dataset had inconsistent flagging. After discussion with the DWI, flagging inconsistencies were resolved for all zones, leaving 170 zones confirmed as ever being fluoridated, of which 7 were noted to have experienced significant disruption to fluoridation operations (see Supplementary Tables 2 and 3 for further details regarding zones with inconsistent flagging).

On linking zones from post-2005 to their pre-2005 counterparts, 13,383 zone years (62% of the total 21,632 pre-2005 zone years) from 3339 zones were not linked. The characteristics of these zones are shown in Table 1 above and can be compared to zone years/zones that were linked between the two periods summarised in Table 2, also above. Fluoridation status could not be assigned to zones that were not linked to any zones from 2006 onwards, when fluoride flagging was initiated. Median fluoride was similar, but slightly lower in the un-linked 1995–2004 zones (0.1 mg/L compared to 0.12 mg/L). There was a similar sampling frequency of just a single annual sample in linked and un-linked zones, and the typical zone duration of 8 years was shorter than linked zones, as expected.

Median fluoride across England of 0.12 mg/L was similar for the two time periods. However, median fluoride was slightly higher for fluoridated WSZs in the latter time period (0.84 mg/L compared to 0.78 mg/L). Most zones linked from the earlier time period contributed data for the entire 1995–2004 monitoring period but were only sampled once a year. Virtually, all of the zones from the latter 2005–2015 time period provided data for 11 years, i.e. they existed since at least 2004.

**Fig. 2:**
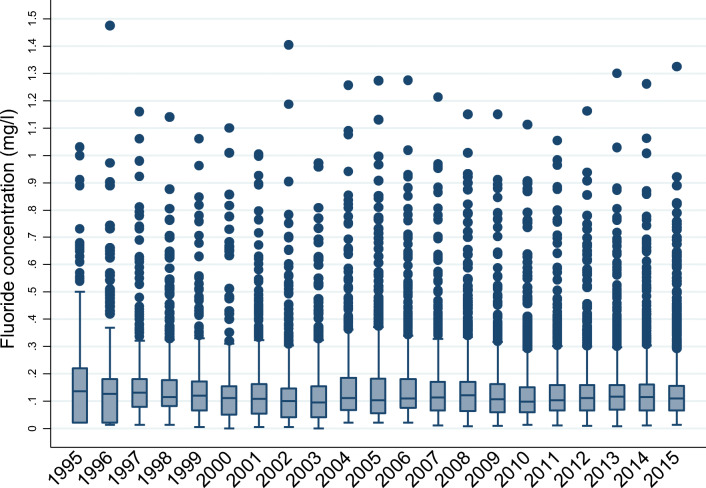
Box plot of annual fluoride concentrations in zones without a fluoridation scheme 1995–2015 (boxes represent values from the 25th to 75th quantiles, the solid horizontal line in each box represents the median value, and dots represent outlying values)

Box plots of annual mean fluoride concentrations from 1995 to 2015 in zones without a fluoridation scheme (see Fig. 2 below) described a relatively stable fluoride concentration across the monitoring period. Apart from 1995, at least 75% of un-fluoridated zones had fluoride concentrations lower than 0.2 mg/L in any year, but there were zones with fluoride concentrations across the range of 0.2 mg/L to maximum concentrations of 1.4–1.5 mg/L. These elevated concentrations likely represented areas with fluoride from geological sources.

**Fig. 3:**
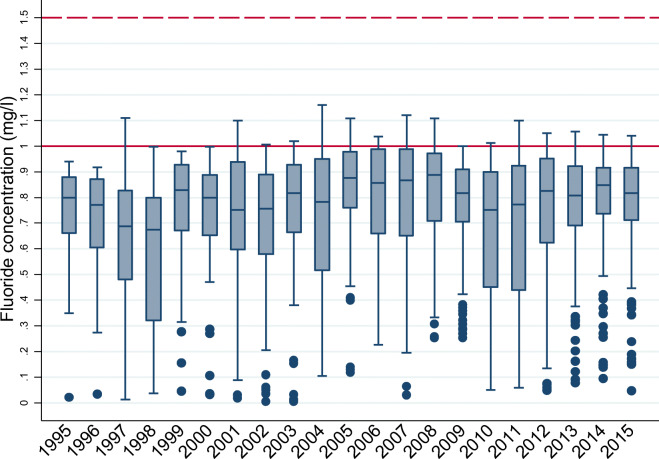
Box plot of annual fluoride concentrations in zones with a fluoridation scheme 1995–2015 (boxes represent values from the 25th to 75th quantiles, the solid horizontal line in each box represents the median value, dots represent outlying values, the solid red horizontal line marks the 1 mg/L target concentration, and the dashed red horizontal line the 1.5 mg/L PCV)

Fluoride concentrations in areas with a scheme (Fig. 3) showed the median of the annual mean fluoride in these areas could fluctuate from as high as 0.9 mg/L to lower than 0.7 mg/L. Seventy-five percent of zones were always at concentrations of < 1 mg/L in each year, and some zones had concentrations of < 0.5 mg/L (fewer than 25% of zones except for 1997, 1998, 2010 and 2011), and as low as < 0.1 mg/L, despite being identified as fluoridated for that year.

**Fig. 4:**
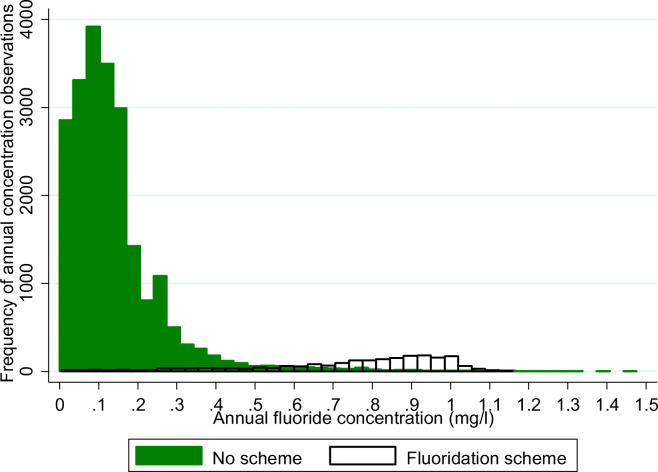
Histogram of annual fluoride concentrations stratified by presence of fluoridation scheme England 1995–2015

The bimodal distribution of annual fluoride concentrations by presence of a scheme is appreciated in Fig. 4. However, there was an overlap in fluoride concentrations in zones with and without a scheme across the range of fluoride concentrations, and the highest concentrations (up to 1.48 mg/L) were noted in zones without a scheme, i.e. where fluoride was present from geological sources.

**Fig. 5:**
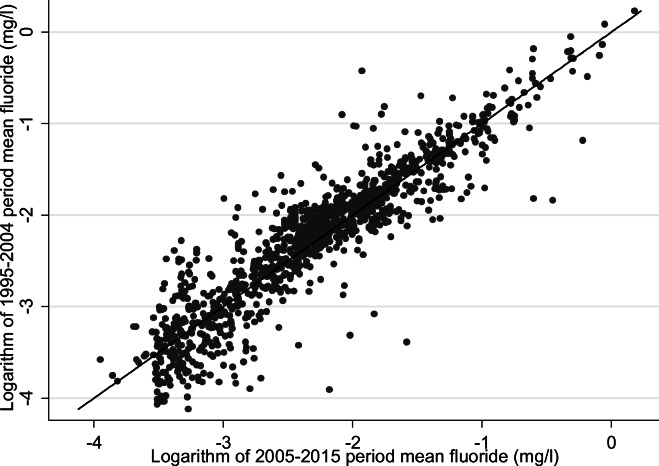
Scatter plot of un-fluoridated* water supply zones comparing 1995–2004 and 2005–2015 period mean fluoride (mg/L) natural log scale with *y* = *x* reference line England 1995–2015^†^. *Bedford Rural, Bedford Urban South, Bedford Urban Central, Ennerdale North, Ennerdale South, Crummock and Crummock South zones excluded from analysis due to identified partial/total non-operation of fluoridation schemes in 2005–2015 period; ^†^For 1424/1477 (1995–2004) and 1825/1878 (2005–2015) unique zones with fluoridation data

**Fig. 6:**
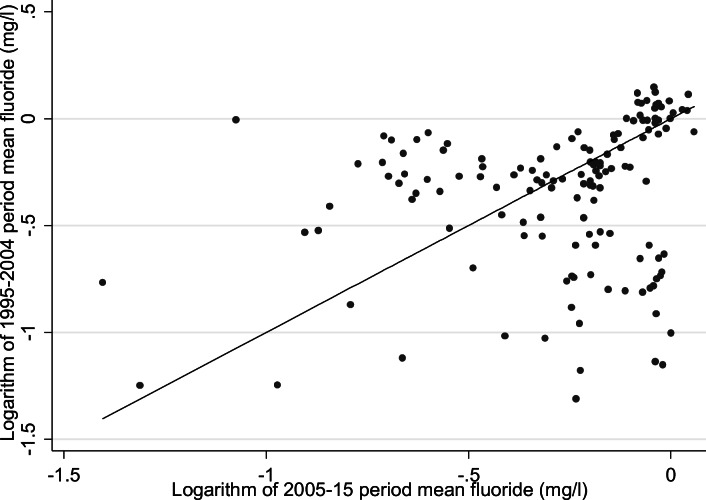
Scatter plot of fluoridated* water supply zones comparing 1995–2004 and 2005–2015 period mean fluoride (mg/L) natural log scale with *y* = *x* reference line England 1995–2015^†^. *Bedford Rural, Bedford Urban South, Bedford Urban Central, Ennerdale North, Ennerdale South, Crummock and Crummock South zones excluded from analysis due to partial/total non-operation of fluoridation scheme; ^†^For 1424/1477 (1995–2004) and 1825/1878 (2005–2015) unique zones with fluoridation data

Comparing aggregated period mean fluoride concentrations (see Figs. 5 and 6), there was a strong correlation (Spearman rank coefficient = 0.93) between period mean fluoride concentrations for the two time periods in un-fluoridated zones.

Summary period fluoride statistics in un-fluoridated zones were very similar across the time periods. The correlation of aggregated period mean fluoride concentrations for fluoridated zones, excluding those where disrupted fluoridation scheme operation was known, was weak (Spearman rank coefficient = 0.31). The latter period fluoride was slightly higher (median 0.84 mg/L compared to 0.78 mg/L) with a narrower interquartile range (0.23 mg/L in 2005–2015 compared to 0.33 mg/L) (Table 3).

**Table 3:** Water supply zone median period fluoride concentration (mg/L) for 1995–2004 and 2005–2015, and Spearman rank coefficient, stratified by fluoridation scheme status, England 1995–2015

Fluoridation scheme^a^	Period	Zones	Range of period fluoride mg/L	Median period fluoride mg/L (LQ–UQ)	Spearman rank coefficient
Yes	1995–2004	141	0.27–1.16	0.78 (0.59–0.92)	–
	2005–2015	161	0.25–1.06	0.84 (0.72–0.95)	0.31
No	1995–2004	1283	0.02–1.26	0.11 (0.06–0.17)	–
	2005–2015	1664	0.01–1.33	0.11 (0.07–0.17)	0.93

Bedford Rural, Bedford Urban South, Bedford Urban Central, Ennerdale North, Ennerdale South, Crummock and Crummock South zones excluded from analysis due to partial/total non-operation of fluoridation scheme

*LQ* lower quartile, *UQ* upper quartile

^a^For 1424/1477 (1995–2004) and 1825/1878 (2005–2015) unique zones with fluoridation data

**Fig. 7:**
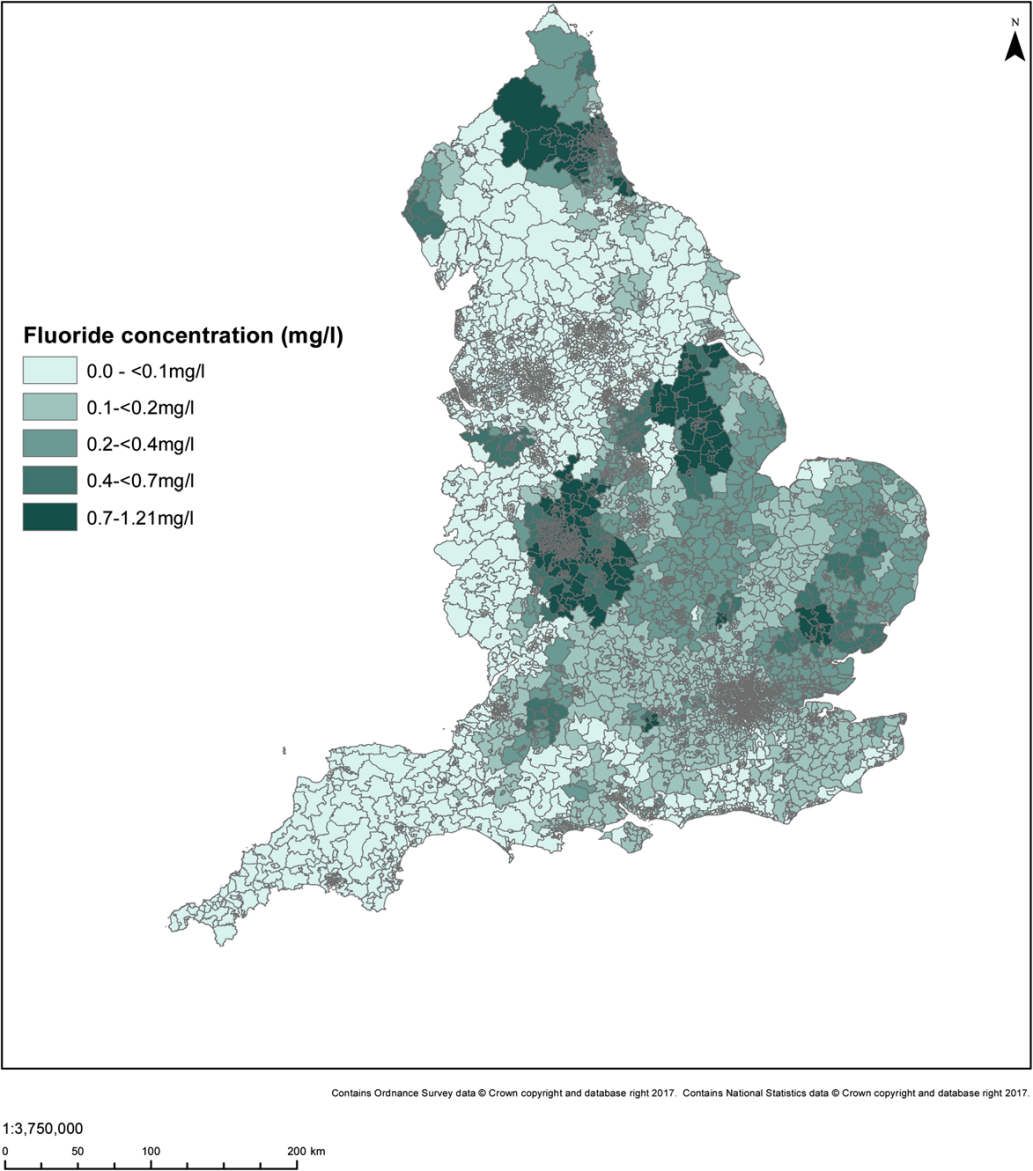
Mean fluoride concentration (mg/L) England 2005–2015 mapped at Middle Layer Super Output Area level using 2011 boundaries

**Fig. 8:**
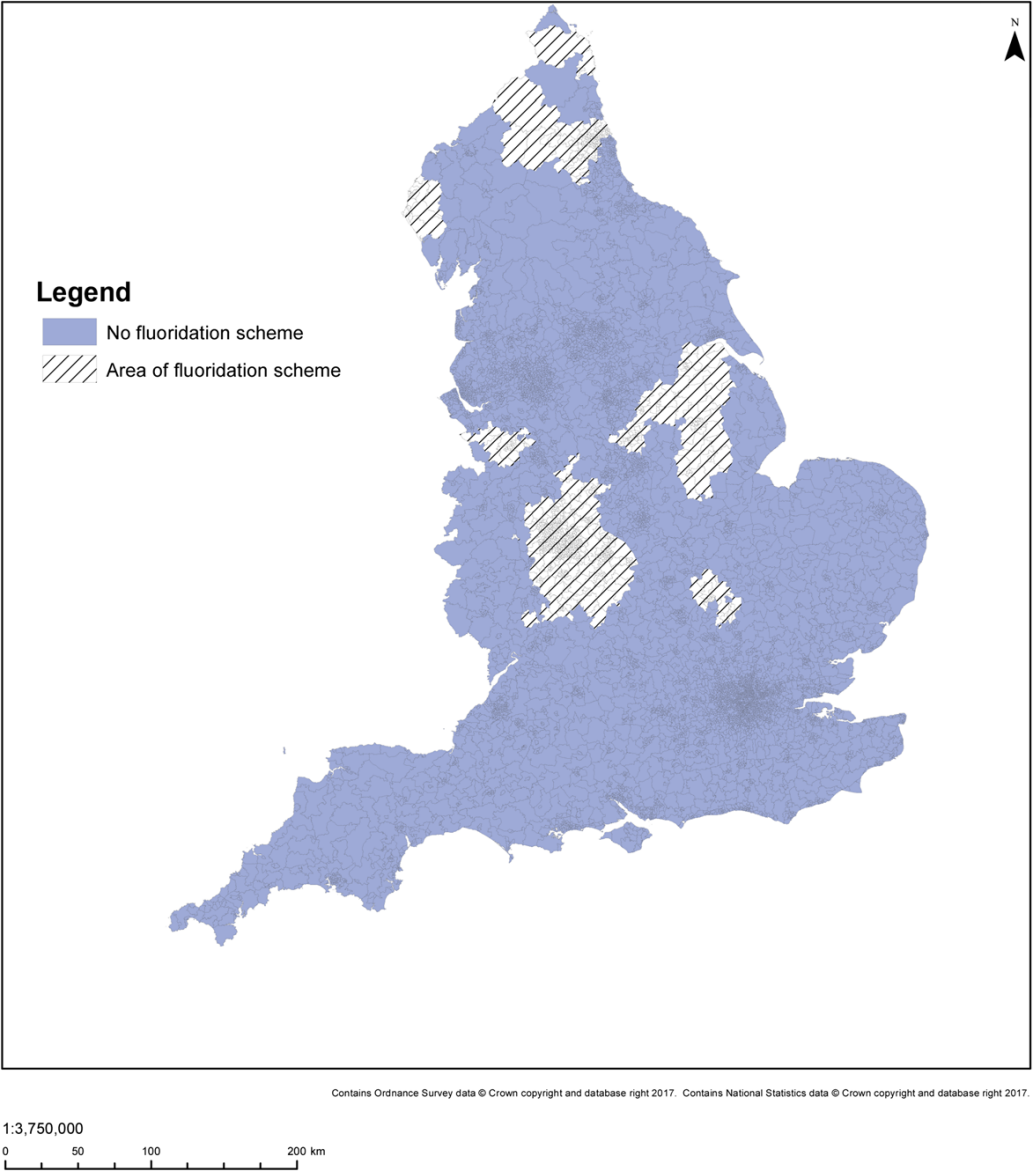
Areas with fluoridation scheme operating at any time during 2005–2015 England mapped at Middle Layer Super Output Area level using 2011 boundaries

The size of populations assumed exposed to different fluoride concentration categories, and to fluoridation schemes, by statistical/administrative geography in England for the 2005–2015 period is summarised below (see Table 4 and Figs. 7 and 8). Fluoride concentrations were not available for the Isles of Scilly, and none were recorded during 2005–2015 for two further LSOAs (both in Richmondshire).

**Table 4:** Number of areas/areas within a fluoridation scheme and average mid-year resident population by period mean fluoride concentration, for different areal units in England 2005–2015

Area unit (2011 boundaries)	Fluoride concentration (mg/L) category	Number of areas (%)	Population^a^ in millions (%)^b^	Number of areas (%): fluoridation scheme^c^	Population^a^ in millions (%)^b^: fluoridation scheme^c^
LSOA	< 0.1 0.1–< 0.2 0.2–< 0.4 0.4–< 0.7 ≥ 0.7 No data Total	12,588 (38) 11,110 (34) 4580 (14) 1302 (4) 3261 (10) 4 (0) 32,844 (100)	19.9 (38) 18.1 (34) 7.3 (14) 2.0 (4) 5.3 (10) 0.0 (0) 52.7 (100)	0 (0) 4 (0) 82 (2) 854 (21) 3065 (77) 0 (0) 4005 (100)	0 (0) 0 (0) 0.1 (2) 1.3 (20) 4.9 (77) 0 (0) 6.4 (100)
MSOA^d^	< 0.1 0.1–< 0.2 0.2–< 0.4 0.4–< 0.7 ≥ 0.7 No data Total	2571 (38) 2317 (34) 957 (14) 280 (4) 665 (10) 1 (0) 6791 (100)	19.6 (37) 18.3 (35) 7.5 (14) 2.2 (4) 5.2 (10) 0.0 (0) 52.7 (100)	0 (0) 0 (0) 20 (2) 185 (22) 625 (75) 0 (0) 833 (100)	0 (0) 0 (0) 0.2 (3) 1.4 (22) 4.9 (77) 0 (0) 6.4 (100)
LTLA^d^	< 0.1 0.1–< 0.2 0.2–< 0.4 0.4–< 0.7 ≥ 0.7 No data Total	107 (33) 115 (35) 62 (19) 19 (6) 22 (7) 1 (0) 326 (100)	18.4 (35) 18.3 (35) 8.5 (16) 2.9 (6) 4.5 (9) 0 (0) 52.7 (100)	0 (0) 0 (0) 2 (6) 11 (32) 21 (62) 0 (0) 34 (100)	0 (0) 0 (0) 0.2 (3) 1.3 (22) 4.4 (75) 0 (0) 5.9 (100)

*LSOA* Lower Layer Super Output Area, *MSOA* Middle Layer Super Output Area, *LTLA* Lower Tier Local Authority

^a^Average mid-year population for 2005–2015

^b^May not sum exactly due to rounding

^c^LSOAs are coded as being supplied by a fluoridation scheme if they have been assigned to a fluoridated water supply zone during 2005–2015, using data supplied by the Drinking Water Inspectorate. MSOAs and LTLAs are defined as fluoridated if at least 50% of their constituent LSOAs were coded as fluoridated

^d^MSOA- and LTLA-level mean fluoride concentration calculated by taking population weighted mean fluoride concentration of constituent LSOAs, using 2005–2015 period population

The particular fluoride concentration, and percentage of areas fluoridated, varies by the different areal units due to aggregation. Most of the population (70% or more) lived in areas where the fluoride concentration in public water supplies was < 0.2 mg/L and 9–10% where it was greater than 0.7 mg/L.

## Discussion

Our analysis of routine fluoride monitoring data detailed how the population of England receives public water supplies with a range of fluoride concentrations, even in areas without fluoridation schemes. Fluoride concentrations in CWF zones were typically lower than target concentrations. We also observed large within-zone annual variation in fluoride concentration in CWF zones and frequently elevated fluoride concentrations in zones without a scheme. Use of water supply boundaries to allocate water fluoridation status has been noted as an efficient method for exposure estimation for public health monitoring studies (Skinner 2012; McLaren 2016), but our findings emphasise the need to use an average of the measured fluoride concentrations. Otherwise, misclassification of exposure may result due to geological sources of fluoride in PWS or variation in CWF performance.

The observed weak correlation between period mean fluoride in fluoridated zones and much stronger correlation in un-fluoridated zones was not unexpected, given fluoride concentration must be maintained within the target range by continuous adjustment by fluoride dosing and/or blending of water supplies. Active fluoridation processes are subject to a range of potential operational challenges such as shortages of fluoride substrate, equipment failure or planned maintenance (though we excluded zones with known major disruption to operation). Limitations in ability to link a large proportion of WSZs across the two time periods mean these results should be treated cautiously, but this poor correlation means there is greater uncertainty in being able to confidently assign a long-term exposure within a narrow concentration range. This would be of most relevance for health outcomes with a likely long lag period from exposure to initiation of pathology, such as cancer and possibly bone fractures, in populations living in areas supplied by fluoridation schemes. Clustering of data points in the bottom right quadrant of the scatterplot (Fig. 6) and the increase in period median fluoride concentration suggest that pre-2005 exposures may be (on average) over-estimated in fluoridated zones if 2005–2015 averages are used as a proxy. In the absence of geo-referenceable data to estimate exposures prior to 2005, using wider fluoride concentration categories may prevent some misclassification, but at the expense of inefficient use of the data and defining a less granular dose response.

Fluoride intake from water depends on both the concentration and the volume of water consumed. While it would be informative to know frequency and quantity of consumption, such data, for example from surveys of drinking water consumption, were only available at a regional level in England; too large a population level to usefully add to the exposure assessment in our study. Exposure duration data would also have been useful but would have required individual residential histories, which were not available and therefore could not be included. It would be interesting to investigate the association between health effects and a measure of the total daily fluoride intake from all sources or the total dose of fluoride absorbed by each person, but again, such total fluoride intake data were not available using routine data. This would also overcome concerns about ‘halo effects’ from dietary manufacturer-added water modifying total fluoride intake. However, as previously discussed, urinary fluoride (a biomarker proxy of dose of fluoride absorbed) strongly correlates with water fluoride concentrations, including at the range of PWS fluoride concentrations observed in our dataset and in a broadly comparable Western population (Till et al. 2018). Further validation of spot urinary fluoride concentration against 24-h urine fluoride as a biomarker of exposure would be helpful to add to the evidence base for their use in adults. However, on the balance of evidence, we consider PWS fluoride concentrations a reasonable indicator of absorbed fluoride dose, particularly once PWS fluoride concentrations increase beyond around 0.3 mg–0.4 mg/L.

Our study has some important limitations. As we have only been able to compare WSZs with stable identifiers over time, we are in effect selecting a sample of WSZs with durable identifiers across the time periods, which may have resulted in differential selection of zones with more/less stable fluoride concentrations. This would only impact on our results if WSZs with stable fluoride concentrations were differentially likely to change ownership (resulting in change in their unique identifier), which would seem unlikely. This is of most concern for unlinked WSZs from 1995 to 2004 zones, as 62% were not linked to 2005–2015 zones for comparison (whereas only 3% of 2005–2015 WSZs with data could not be used in this analysis as they could not be linked to map data). However, the median fluoride concentrations and fluoride sampling frequency were similar for both linked and un-linked 1995–2004 WSZs, giving more confidence in our findings. We analysed routine fluoride concentration monitoring data collated for water quality verification; the sampling method was not designed for the purposes of health monitoring, which brings limitations. Though sample points were randomly chosen or selected so as to be representative of the wider WSZ, precision (due to sampling frequency) and accuracy (due to the location of sampling and/or measurement methods used) are likely to have been less optimal than could be achieved from a survey designed specifically for research purposes. However, the long time periods of data collection and relatively uniform sampling procedures used will have negated some of these concerns. We were also reassured by the frequency and consistency of concentrations < 0.15 mg/L (the mandated lower detection limit) in zones without schemes or geological fluoride sources, matching expectations from previous work which described PWS fluoride concentrations in England and Scotland (Blakey et al. 2014) that operational testing outperforms this minimum standard. Additionally, communities may have had their PWS fluoride concentrations misclassified if WSZ digital boundaries poorly circumscribed the source of water supplied. PWS fluoride concentrations derived using our methods did correlate with less prevalent and less severe caries in an approximately dose-dependent manner on health monitoring (Public Health England 2018), meaning there is some face validity, but biomarker studies to validate the routine monitoring data could be considered. The number of annual monitoring samples increased in 2005–2015 compared to 1995–2004 (to a median of 8 from a median of 1), indicating a change in the frequency of fluoride concentration monitoring that coincided with the introduction of new regulations. This or other changes which we have not measured may have introduced misclassification if this resulted in a change in precision of WSZ fluoride concentration estimation across the two time periods. When comparing fluoride concentrations in WSZs with and without a scheme between 1995 and 2004 and 2005–2015, we only excluded WSZs where disruption to fluoridation was detected by inconsistent flagging of fluoridated WSZs reported to DWI by water companies. This is likely to not take into account shorter term disruption, potentially weakening correlations across the time periods for fluoridated WSZs if disruption varied by time period.

In conclusion, we were able to use routine PWS fluoride concentration monitoring data to estimate population PWS fluoride exposure in England. These estimates provide an efficient method for estimating exposure for public health monitoring and are more accurate than using fluoridation status alone. Additionally, the use of exposure categories allows the detection of an exposure-outcome dose–response which can aid epidemiological inference of causation. Including low PWS fluoride concentrations in these categories allows monitoring of health effects potentially present only at lower exposure concentrations. However, such associations should be interpreted cautiously, as PWS fluoride concentrations are more likely to provide a reasonable proxy for total fluoride exposure at higher PWS fluoride concentrations (e.g. above 0.3–0.4 mg/L), when fluoride intake from water will make up a greater proportion of the total intake. The 1995–2004 and 2005–2015 period mean WSZ fluoride concentrations were similar, but concentrations were more stable in WSZs without CWF. This means fluoride exposure prior to availability of geo-referenceable PWS fluoride monitoring data in 2005 could be estimated using data from the later period, but there is a greater risk of misclassification of 1995–2004 fluoride concentrations in CWF zones.

## Electronic supplementary material

ESM 1 (DOCX 14 kb)

## Footnotes

1 Each water supply zone may contribute varying durations of concentration data; therefore, one ‘zone year’ is equivalent to 1 year of fluoride concentration data for one water supply zone.
