# Supplementary material for: Use of public water supply fluoride concentration as an indicator of population exposure to fluoride in England 1995–2015
Source: Environ Monit Assess. 2020 Jul 14;192(8):514. doi: 10.1007/s10661-020-08304-3 (PMC7360650; doi:10.1007/s10661-020-08304-3)
Supplement: Supplementary file 1 — (DOCX 14 kb) [file 10661_2020_8304_MOESM1_ESM.docx]

**Supplementary table 1. Characteristics of geographic units of analysis(1)**

| **Unit of analysis** | **Acronym** | **Number in England** | **Average population 2011** |
| --- | --- | --- | --- |
| Lower layer Super Output Area 2011 | LSOA | 32,844 | 1,614 |
| Middle layer Super Output Area 2011 | MSOA | 6,791 | 7,806 |
| Lower Tier Local Authority* | LTLA | 326 | 162,615 |

*Also known as local authority district

1. Office for National Statistics. Census geography London [Available from: <https://www.ons.gov.uk/methodology/geography/ukgeographies/censusgeography#super-output-area-soa>.

**Supplementary table 2.** Resolution of WSZ fluoridation flagging inconsistencies, England 2006-2015

| Reason for inconsistency | Count |
| --- | --- |
| False positive in 1 or more years i.e. never fluoridated | 22 |
| False negative in 1 or more years i.e. fluoridated | 11 |
| Total | 33 |

**Supplementary table 3.** Water supply zones with significant disruption to operations, England 2006-2015

| **Zone code** | **Region** | **Years affected** |
| --- | --- | --- |
| ANGZMW23 | Bedford Rural | 2009-2015 |
| ANGZMW25 | Bedford Urban South | 2009-2015 |
| ANGZMW26 | Bedford Urban Central | 2009-2015 |
| UUTZ031 | Ennerdale North | 2012-2013 |
| UUTZ032 | Ennerdale South | 2012-2013 |
| UUTZ028 | Crummock | 2007-2013 |
| UUTZ029 | Crummock South | 2007-2009 |
